# Relationships between nutritional intake, appetite regulation, and mental health with body composition among female college students with overweight and obesity

**DOI:** 10.3389/fpsyg.2025.1465784

**Published:** 2025-05-14

**Authors:** Qiang Wang, Soh Kim Geok, Mahmoud Danaee, Wan Ying Gan, Wang Li Zhu, Yi Qiang Mai, Sheng Yao Luo

**Affiliations:** ^1^Department of Sport Studies, Faculty of Educational Studies, Universiti Putra Malaysia, Seri Kembangan, Malaysia; ^2^Faculty of Physical Education, Yichun Early Childhood Teacher College, Yichun, China; ^3^Department of Social and Preventive Medicine, Faculty of Medicine, University of Malaya, Kuala Lumpur, Malaysia; ^4^Department of Nutrition and Dietetics, Faculty of Medicine and Health Sciences, Universiti Putra Malaysia, Seri Kembangan, Malaysia; ^5^Institute of Education Development, Nanchang University, Nanchang, China; ^6^School of Physical Education and Taijiquan, Henan Polytechnic University, Jiaozuo, China; ^7^Faculty of Physical Education and Art, Jiangxi University of Science and Technology, Ganzhou, China

**Keywords:** overweight, obesity, college students, body composition, nutritional intake, appetite regulation, mental health

## Abstract

**Introduction:**

The prevalence of overweight and obesity among college girls is a significant public health concern. This cross-sectional study investigated the relationships between nutritional intake, appetite regulation, and mental health with body composition among overweight and obese college girls.

**Methods:**

This study involved 72 college girls. Standardized instruments measured the corresponding variables. The data analysis utilized Pearson and Spearman correlations.

**Results:**

Results show that energy and carbohydrate intake were positively correlated with body fat percentage and waist circumference (both *p* ≤ 0.007). Fat intake was positively correlated with all body composition variables (all *p* < 0.001). Anxiety was negatively correlated with all body composition variables (all *p* ≤ 0.027). Hunger at 0 min was positively correlated with body fat percentage and waist circumference (both *p* ≤ 0.002). Hunger at 60 min was positively correlated with BMI and waist circumference (both *p* ≤ 0.012). Desire to eat at 0 and 60 min were positively correlated with all body composition variables (all *p* ≤ 0.003). Desire to eat at 30 min was positively correlated with BMI (*p* = 0.005). Desire to eat at 90 min was negatively correlated with body fat percentage (*p* = 0.047). Fullness at 0 min was positively correlated with waist circumference (*p* = 0.040). Fullness at 30 min was positively correlated with body fat percentage and waist circumference (both *p* ≤ 0.018). Fullness at 120 min was negatively correlated with all body composition variables (all *p* ≤ 0.023). Prospective food consumption at 0 min was positively correlated with all body composition variables (all *p* < 0.001). Prospective food consumption at 30, 60, and 120 min was positively correlated with BMI (all *p* ≤ 0.008).

**Discussion:**

Overall, overweight and obese college girls should manage energy intake, fat intake, carbohydrate intake, anxiety, and appetite regulation to reduce fat levels. Further research suggests exploring counterintuitive correlations between body composition with anxiety, desire to eat at 90 min, and fullness at 0 and 30 min, along with limitations related to causal relationships, measurement accuracy, the relationship with physical activity, and population diversity.

## 1 Introduction

According to the latest WHO global estimates, 2.5 billion adults aged 18 years and older were overweight (43%), with nearly 890 million living with obesity (16%). The global age-standardized prevalence of obesity increased from 8.8% in 1990 to 18.5% in 2022 for women and from 4.8% to 14.0% for men (Phelps et al., 2024). In 2022, there were an estimated 44% women and 43% men living with overweight globally (Okunogbe et al., 2022). Women have higher overweight and obesity rates than men, indicating that women face a greater risk and burden of fat-related health problems.

The high prevalence of overweight and obesity among female college students is a significant public health concern that requires immediate attention. A recent study in Ghana by Obirikorang et al. (2024) showed that the prevalence of obesity was higher in female college students (9.4%) than male students (6.7%). The findings further revealed that weight status indicated abnormal central and general adiposity, along with high body fat accumulation, in 17.0% of male students and 33.8% of female students. A review reported that weight gain in young women aged 18–35 years was greater than in older women (Pegington et al., 2020). Several factors contribute to the high rates of overweight and obesity among female college students. Erratic eating patterns, often influenced by demanding academic schedules, can lead to poor dietary choices (Choi, 2020). In addition, high stress levels associated with educational and social pressures can further exacerbate the problem (Jiang et al., 2018). Furthermore, limited access to healthy food options on university campuses can make it difficult for students to maintain a balanced diet. The consequences of overweight and obesity among female college students are far-reaching and potentially life-threatening. These conditions significantly increase the risk of developing a variety of health problems, including metabolic syndrome, adverse cardiovascular events, non-alcoholic fatty liver disease, and cancer, thereby endangering the lives of the population with obesity (Brehm and Steffen, 2013; Faris et al., 2022; Bhattarai et al., 2023).

According to the first law of mechanics and the dietary balance model, the quality and quantity of food consumed directly influence body weight (Walsh, 2013; Carreiro et al., 2016). In college environments, where students often experience newfound independence from parental dietary supervision, there is a greater tendency to consume calorie-dense but nutrient-poor foods (Mancone et al., 2024). This dietary pattern can lead to increased caloric intake, significant weight gain, and altered metabolic pathways, posing significant challenges for weight management (French et al., 2000; Arciero et al., 2013).

On the contrary, being overweight or obese can significantly alter eating behaviors. This change is partly due to hormonal changes that affect physiological hunger cues. For instance, obesity can increase levels of ghrelin (the hunger hormone), leading to increased calorie intake, often from foods that are energy-dense yet nutrient-poor (Cummings et al., 2002; Myers et al., 2010). Furthermore, psychological factors such as stress, which is prevalent among overweight and obese students, often exacerbate unhealthy eating habits (Reichenberger et al., 2021).

Changes in perceived appetite can have a significant impact on an individual's body fat levels. Research has shown that higher perceived feelings of hunger and a stronger desire to eat (DTE) tend to increase overall caloric intake (French et al., 2000; Blundell et al., 2015). When individuals experience heightened hunger and a greater desire to eat, they may consume larger portions or eat more frequently in an attempt to achieve a sense of satiety. However, these altered appetite cues can lead to a higher energy intake than what is actually required by the body. This imbalance between energy intake and expenditure can perpetuate or exacerbate weight gain over time (Bryant et al., 2008; Rosenbaum et al., 2010).

Meanwhile, obesity alters the body's appetite regulation system. Increased body fat can lead to hormonal imbalances, such as leptin resistance; despite high levels of leptin, which should signal satiety, the body continues to feel hungry. This hormonal disruption leads to a decreased sensation of fullness and a continuous DTE (Schwartz et al., 2000; Cummings et al., 2002). According to the satiety cascade theory, perceived appetite includes hunger, satiety, expected food consumption, and DTE (Mela, 2006). However, current research on overweight and obese individuals often focuses on only one or two of these perceived appetite variables (Larsen et al., 2015; Sari et al., 2021; Taş and Gezer, 2022). To gain a comprehensive understanding of the relationship between body composition and appetite regulation in overweight and obese female college students, it is necessary to investigate all perceived appetite variables of the satiety cascade theory in this study.

Furthermore, according to the stress-eating hypothesis, mental health conditions such as depression, anxiety, and stress can also indirectly influence an individual's fat level through their eating behaviors and energy intake (Geiker et al., 2018). Psychological distress often leads to emotional eating, where people use food as a means of self-soothing. This can lead to increased caloric intake, particularly of high-fat and high-sugar comfort foods that are less satiating and nutritionally poor. This type of eating behavior not only contributes to maintaining and exacerbating obesity but also disrupts metabolic responses, making weight management even more challenging (Gibson, 2006; Luppino et al., 2010). By understanding the complex interplay between mental health, eating behavior, and body composition, researchers and healthcare providers can develop more comprehensive and effective interventions to address the multiple needs of overweight and obese female college students.

It is important to note that the relationship between weight status and mental health is bidirectional. Not only can mental health conditions affect eating behavior and body composition, but being overweight or obese can also exacerbate mental health issues (Saghiv and Sagiv, 2020). The stigma associated with excess weight can lead to social isolation, increased stress, and lower self-esteem, all of which can heighten symptoms of depression and anxiety. Moreover, the physiological impacts of obesity, such as hormone imbalances and inflammation, may further contribute to psychological distress. This creates a vicious cycle where mental health problems and obesity perpetuate each other, complicating treatment and intervention strategies (Lloyd-Richardson et al., 2009; Luppino et al., 2010).

Therefore, in order to effectively break these cycles, intervention measures must address both mental health and dietary behavior issues. However, a systematic review by Vajdi and Farhangi (2020) discussed the gaps in research focusing on the interplay between dietary behavior, mental health, and body composition, highlighting that most studies tend to examine these variables in isolation rather than in conjunction. Furthermore, meta-analyses by Psaltopoulou et al. (2013) and Teasdale et al. (2019) noted that while there is a significant body of research examining the interaction between dietary patterns and mental health, the simultaneous assessment of how dietary behaviors and mental health factors affect body composition is still relatively underexplored and needs more comprehensive research. In addition, research in this area has often generalized findings from mixed-gender populations, neglecting the unique physiological and psychological factors that contribute to females (Haynes et al., 2018; Kantowski et al., 2024). Similarly, studies focusing on college students have not adequately addressed the particular challenges faced by this demographic, often grouping them with broader adult populations (Haidar et al., 2018; Lee et al., 2021). Another meta-analysis showed that the rate of energy intake underreporting is about 10% higher in overweight and obese populations compared to the average, indicating a need for more precise studies in this demographic (Teasdale et al., 2019). The findings of this study will serve as a theoretical foundation for the development of multidisciplinary team weight loss intervention strategies that aim to enhance the physical and mental health of female college students in the future.

## 2 Methods

### 2.1 Participants and design

During March 2024, 152 overweight and obese (BMI > 25) female college students were enrolled for the assessment of eligibility from the Yichun Early Childhood Teacher College (79 samples) and Yichun College (73 samples) in Yichun, Jiangxi, China, as research subjects through leaflets and conference promotion. The subjects should be healthy and have previously lacked a systematic weight loss plan, including regular and active exercise programs in the last 6 months as well as systematic diets for the last 3 months (Beigrezaei et al., 2021). Participants should not have an irregular menstrual cycle or an abnormal menstrual cycle (Chung, 2007). Furthermore, researchers exclude participants who refuse to comply with the specified measurement procedures. Researchers utilize the G^*^ power and effective sample size equation to determine the necessary sample size (Killip et al., 2004; Shaw et al., 2010; Brysbaert and Stevens, 2018). Finally, researchers randomly included 86 (43 samples in each college) students in the study using a computer-generated random sequence. All participants have received a brief introduction and guidance on the measurement procedures.

During the data collection period of this cross-sectional study, the researchers asked the participants to maintain their previous lifestyle, refraining from high-intensity and high-dose activities, avoiding intentional accidents that could trigger overstimulating emotions, and avoiding overeating and dieting. The researchers did not instruct the subjects to alter individuals' body composition, nutritional intake, appetite regulation, or mental health. Therefore, this study provides a unique opportunity to observe the natural associations between body composition and other variables. Researchers performed all methods in accordance with the relevant guidelines and regulations.

### 2.2 Measurement

#### 2.2.1 Body composition

The DHM-301w ultrasonic measuring device from Zhengzhou Dingheng Electronic Technology Company measured body weight and height to calculate BMI (Peterson et al., 2016; Allman, 2018). Researchers assessed body fat percentage by measuring the skinfold in the triceps, thigh, and supra-iliac using the Idass Company's Harpenden skinfold caliper model C-136, in conjunction with the Jackson-Pollock equation and the Siri equation (Guerra et al., 2010; Klesges et al., 2010; Gomes et al., 2022). Waist circumference was measured using the inelastic fiberglass measuring tape with the KOMELON brand KMC-330 (Song and Oh, 2024). All body composition devices have precise accuracy, durable construction, and user-friendly design. Based on the conducted analysis, the three devices exhibited excellent test-retest reliabilities (ICC = 0.999, 0.993, and 0.992, following the above order), while very strong validities were reported in the respective device introductions (*r* = 0.98, 0.94, and 0.97, in the same order; Mason and Katzmarzyk, 2009; Chen, 2022; Cintra-Andrade et al., 2023).

#### 2.2.2 Nutrition intake

Researchers instructed the participants to record their food intake for 3 days, which included 2 weekdays and 1 weekend day. This 3-day food record collected data on energy intake, protein intake, fat intake, and carbohydrate intake with acceptable reliability (ICC = 0.48–0.69) and strong validity (*r* = 0.66–0.80; Putz et al., 2019). Researchers performed the dietary analysis using Elizabeth Stewart Hands and Associates' Food Processor Nutrition Analysis and Fitness Software (ESHA, Version11.12.X, 2022), an extensive and reliable food composition database (Bales et al., 2012). Following analysis, researchers averaged the nutrition intake over the 3 days to determine the daily values (Kolar et al., 2005).

#### 2.2.3 Appetite regulation

A 100-mm visual analog scale for appetite assessment was administered between 7:00 and 9:00 AM, following a 10-h overnight fast. Participants first rated their perceived appetite. Then they immediately received a standardized caloric stimulus produced by the CHUNDUBIO Company. Appetite ratings were then assessed again at 30, 60, 90, and 120 min after consumption. The visual analog scale included four domains: DTE, hunger, fullness, and prospective food consumption (PFC), which has good reliability (ICC = 0.72–0.98) and strong validity (*r* = 0.56–0.63; Bellissimo et al., 2007, 2008; Hammond et al., 2022).

#### 2.2.4 Mental health

This study used the Depression, Anxiety, and Stress Scale (DASS-21) to measure the participants' levels of depression, anxiety, and stress (Cao et al., 2023). It consisted of 21 items, with 7 items each for depression, anxiety, and stress. Participants rated the extent to which they had experienced each state over the past week on a 4-point scale from 0 (did not apply to me at all) to 3 (applied to me very much or most of the time). The DASS-21 has demonstrated good psychometric properties, including reliability (Cronbach α = 0.76–0.89 and ICC = 0.72–0.80) and validity (*r* = 0.531–0.598), across diverse populations and settings (Gong et al., 2010; Nada et al., 2022).

#### 2.2.5 Physical activity and basic information

Exercise behavior was assessed using the Physical Activity Stages of Change questionnaire, which categorizes individuals into five stages: precontemplation, contemplation, preparation, action, and maintenance (Marshall and Biddle, 2001). Students in precontemplation and contemplation stages (stages 1 and 2) were selected according to exercise criteria. The Chinese adaptation of the questionnaire has demonstrated good reliability (Cronbach α 0.71 and ICC 0.89–0.93) and high concurrent validity of the group method (Abula et al., 2016; Farmanbar et al., 2018). In addition, a brief questionnaire was designed to gather participants' basic information and details regarding other exclusion criteria.

### 2.3 Data analysis

All the data were analyzed using IBM SPSS Statistics for Windows Version 27.0. The Kolmogorov-Smirnov and Shapiro-Wilk tests were utilized to ascertain whether the data were normally distributed. The outcomes of both tests indicated that most of the study variables followed a normal distribution. To ensure data integrity and reliability, the researcher used both the Box Plot and Z-Score methods to detect potential outliers in the dataset, indicating that there are no significant outliers. Additionally, the researcher described the continuous data using the mean and standard deviation. Inferential statistics were used. The Pearson-product-moment correlation test was used to determine the relationship between continuous variables if the data were normally distributed, while the Spearman test was used if the data were not normally distributed. The Bonferroni correction was used to adjust the *p*-values, ensuring a more stringent threshold for statistical significance. The significance value was set at *p* < 0.05 (Garson, 2012; Byrne, 2013; Meyers et al., 2016). The Supplementary material lists the results of normal distributions, descriptive statistics, Z-scores, and box plots of variables.

## 3 Results

### 3.1 Participants characteristics

A total of 86 participants' information was collected, and 72 (38 samples in Yichun Early Childhood Teacher College; 34 samples in Yichun College) participants' information was used for the final analysis. Whereas, 14 copies that could not be used for analysis owing to insufficient data were excluded (Table 1). The participants' mean age was 19.93 years, and their mean BMI was 28.64 kg/m2. The mean energy, protein, fat, and carbohydrate intake was 2156.64 kcal, 80.50 g, 70.12 g, and 301.17 g. Specifically, a total of 27 (37.5%), 23 (31.9%), and 22 (30.6%) of the sample were in their 1st, 2nd, and 3rd years of college.

**Table 1:** Participants' characteristics (*n* = 72).

**Variables**	**Mean ±SD**	**Range**	***n* (%)**
Age (years)	19.93 ± 0.92	18.31–21.72	
Grade			
Freshman			27 (37.5%)
Sophomore			23 (31.9%)
Junior			22 (30.6%)
BMI (kg/m^2^)	28.64 ± 3.23	25.02–33.66	
Energy intake (kcal)	2156.64 ± 289.24	1655.31–2733.99	
Protein intake (g)	80.50 ± 21.22	44.27–116.89	
Fat intake (g)	70.12 ± 20.04	40.04–101.66	
Carbohydrate intake (g)	301.17 ± 61.39	206.46–393.79	

### 3.2 Relationship between nutritional intake and body composition

Table 2 shows that fat intake was significantly and positively correlated with BMI moderately (*r* = 0.460, *p* < 0.001), body fat percentage strongly (*r* = 0.608, *p* < 0.001), and waist circumference strongly (*r* = 0.648, *p* < 0.001). Energy intake was significantly and positively correlated with body fat percentage (*r* = 0.591, *p* < 0.001) and waist circumference (*r* = 0.552, *p* < 0.001) moderately. Carbohydrate intake was significantly and positively correlated with body fat percentage (*r* = 0.320, *p* = 0.006) and waist circumference (*r* = 0.317, *p* = 0.007) weakly, but not protein intake.

**Table 2:** Correlation of nutritional intake and body composition.

**Variables**	**BMI**		**Body fat percentage**		**Waist circumference**	
	***r***	***P*** **-value**	***r***	***P*** **-value**	***r***	***P*** **-value**
Energy intake	0.227^b^	0.055	0.591^**a^	< 0.001	0.552^**b^	< 0.001
Protein intake	0.073^b^	0.543	0.136^b^	0.255	0.195^b^	0.100
Fat intake	0.460^**b^	< 0.001	0.608^**a^	< 0.001	0.648^**b^	< 0.001
Carbohydrate intake	0.211^b^	0.075	0.320^**b^	0.006	0.317^**b^	0.007

^a^Spearman correlation, ^b^Pearson correlation.

^**^Correlation is significant at the 0.01 level (2-tailed).

### 3.3 Relationship between mental health and body composition

Table 3 shows that anxiety was significantly and negatively correlated to BMI (*r* = −0.283, *p* = 0.016), body fat percentage (*r* = −0.273, *p* = 0.020), and waist circumference (*r* = −0.261, *p* = 0.027) weakly, but not depression and stress.

**Table 3:** Correlation of mental health and body composition.

**Variables**	**BMI**		**Body fat percentage**		**Waist circumference**	
	***r***	***P*** **-value**	***r***	***P*** **-value**	***r***	***P*** **-value**
Depression	−0.110*^*b*^*	0.358	−0.212*^*b*^*	0.074	−0.176*^*b*^*	0.140
Anxiety	−0.283^*b^	0.016	−0.273^*a^	0.020	−0.261^*b^	0.027
Stress	−0.042*^*b*^*	0.724	0.026^a^	0.827	0.114*^*b*^*	0.340

^a^Spearman correlation, ^b^Pearson correlation.

^*^Correlation is significant at the 0.05 level (2-tailed).

### 3.4 Relationship between hunger and body composition

Table 4 shows that hunger at 0 min was significantly and positively correlated with body fat percentage (*r* = 0.375, *P* = 0.001) and waist circumference (*r* = 0.351, *P* = 0.002) weakly. Hunger at 60 min was significantly and positively correlated with BMI (*r* = 0.293, *P* = 0.012) and waist circumference (*r* = 0.324, *P* = 0.006) weakly, but not hunger at 30, 90, and 120 min.

**Table 4:** Correlation of body composition and hunger at five timepoints.

**Variables**	**BMI**		**Body fat percentage**		**Waist circumference**	
	***r***	***P*** **-value**	***r***	***P*** **-value**	***r***	***P*** **-value**
Hunger 0 min	0.128*^*b*^*	0.284	0.375^**a^	0.001	0.351^**b^	0.002
Hunger 30 min	0.084*^*b*^*	0.485	−0.154^a^	0.196	−0.209*^*b*^*	0.078
Hunger 60 min	0.293^*b^	0.012	0.222*^*b*^*	0.061	0.324^**b^	0.006
Hunger 90 min	0.158*^*b*^*	0.185	−0.059^a^	0.621	−0.039*^*b*^*	0.746
Hunger 120 min	0.201*^*b*^*	0.091	−0.172*^*b*^*	0.149	−0.087*^*b*^*	0.467

^a^Spearman correlation, ^b^Pearson correlation.

^*^Correlation is significant at the 0.05 level (2-tailed).

^**^Correlation is significant at the 0.01 level (2-tailed).

### 3.5 Relationship between DTE and body composition

Table 5 shows that DTE at 0 min was positively and significantly correlated with BMI (*r* = 0.504, *P* < 0.001) moderately, as well as body fat percentage (*r* = 0.388, *P* = 0.001) and waist circumference (*r* = 0.371, *P* = 0.001) weakly. DTE at 30 min was positively and significantly correlated with BMI weakly (*r* = 0.331, *P* = 0.005). DTE at 60 min was positively and significantly correlated with BMI (*r* = 0.528, *P* < 0.001) moderately, as well as body fat percentage (*r* = 0.375, *P* = 0.001) and waist circumference (*r* = 0.343, *P* = 0.003) weakly. DTE at 90 min was negatively and significantly correlated with body fat percentage weakly (*r* = −0.235, *P* = 0.047), but not DTE at 120 min.

**Table 5:** Correlation of body composition and DTE at five timepoints.

**Variables**	**BMI**		**Body fat percentage**		**Waist circumference**	
	***r***	***P*** **-value**	***r***	***P*** **-value**	***r***	***P*** **-value**
DTE 0 min	0.504^**b^	< 0.001	0.388^**b^	0.001	0.371^**b^	0.001
DTE 30 min	0.331^**b^	0.005	0.144^a^	0.228	0.063*^*b*^*	0.598
DTE 60 min	0.528^**b^	< 0.001	0.375^**a^	0.001	0.343^**b^	0.003
DTE 90 min	0.063*^*b*^*	0.601	−0.235^*b^	0.047	−0.176*^*b*^*	0.140
DTE 120 min	0.173*^*b*^*	0.146	−0.194*^*b*^*	0.102	−0.132*^*b*^*	0.271

^a^Spearman correlation, ^b^Pearson correlation.

^*^Correlation is significant at the 0.05 level (2-tailed).

^**^Correlation is significant at the 0.01 level (2-tailed).

### 3.6 Relationship between fullness and body composition

Table 6 shows that fullness at 0 min was positively and significantly correlated with waist circumference weakly (*r* = 0.242, *P* = 0.040). Fullness at 30 min was positively and significantly correlated with body fat percentage (*r* = 0.277, *P* = 0.018) and waist circumference (*r* = 0.305, *P* = 0.009) weakly. Fullness at 120 min was negatively and significantly correlated with BMI (*r* = −0.346, *P* < 0.001), body fat percentage (*r* = −0.267, *P* = 0.023), and waist circumference (*r* = −0.312, *P* = 0.008) weakly, but not fullness at 60 and 90 min.

**Table 6:** Correlation of body composition and fullness at five timepoints.

**Variables**	**BMI**		**Body fat percentage**		**Waist circumference**	
	***r***	***P*** **-value**	***r***	***P*** **-value**	***r***	***P*** **-value**
Fullness 0 min	0.070*^*b*^*	0.561	0.155*^*b*^*	0.195	0.242^*b^	0.040
Fullness 30 min	−0.057*^*b*^*	0.632	0.277^*b^	0.018	0.305^**b^	0.009
Fullness 60 min	−0.101*^*b*^*	0.399	0.206^a^	0.082	0.132*^*b*^*	0.269
Fullness 90 min	−0.094*^*b*^*	0.433	0.188^a^	0.114	0.068*^*b*^*	0.571
Fullness 120 min	−0.346^**b^	0.003	−0.267^*b^	0.023	−0.312^**b^	0.008

^a^Spearman correlation, ^b^Pearson correlation.

^*^Correlation is significant at the 0.05 level (2-tailed).

^**^Correlation is significant at the 0.01 level (2-tailed).

### 3.7 Relationship between PFC and body composition

Table 7 shows that the PFC at 0 min was positively and significantly correlated with BMI (*r* = 0.405, *P* < 0.001), body fat percentage (*r* = 0.459, *P* < 0.001), and waist circumference (*r* = 0.486, *P* < 0.001) moderately. PFC at 30 (*r* = 0.319, *P* = 0.006), 60 (*r* = 0.308, *P* = 0.008), and 120 min (*r* = 0.332, *P* = 0.004) were positively and significantly correlated with BMI weakly, but not PFC at 90 min.

**Table 7:** Correlation of body composition and PFC at five timepoints.

**Variables**	**BMI**		**Body fat percentage**		**Waist circumference**	
	***r***	***P*** **-value**	***r***	***P*** **-value**	***r***	***P*** **-value**
PFC 0 min	0.405^**b^	< 0.001	0.459^**b^	< 0.001	0.486^**b^	< 0.001
PFC 30 min	0.319^**b^	0.006	0.103*^*b*^*	0.387	0.058*^*b*^*	0.629
PFC 60 min	0.308^**b^	0.008	0.067*^*b*^*	0.577	0.089*^*b*^*	0.457
PFC 90 min	0.230*^*b*^*	0.052	0.017*^*b*^*	0.885	0.070*^*b*^*	0.557
PFC 120 min	0.332^**b^	0.004	0.196*^*b*^*	0.099	0.183*^*b*^*	0.124

^a^Spearman correlation, ^b^Pearson correlation.

^**^Correlation is significant at the 0.01 level (2-tailed).

## 4 Discussion

This finding suggests that energy intake, fat intake, carbohydrate intake, anxiety, and perceived appetite at different timepoints have significant correlations with body composition among overweight and obese female college students. Specifically, high fat intake correlates with increased BMI, body fat percentage, and waist circumference, suggesting that higher intake of fat was associated with increased body composition among those overweight and obese female college students. This is consistent with research by Malik et al. (2006), which analyzed data from young adults and found that saturated fat intake was particularly impactful on body composition. The reason is that high fat intake, particularly saturated fats, leads to increased body fat and abdominal obesity due to higher caloric density and metabolic effects (Malik et al., 2013). Furthermore, the findings indicate a significant positive correlation between energy intake with both body fat percentage and waist circumference, consistent with previous research. Du et al. (2010) found that higher energy consumption was associated with increased body fat and abdominal obesity in a cohort of Europeans aged 20–70 years. Similarly, the significant positive correlation between carbohydrate intake with body fat percentage and waist circumference aligns with Myles (2014), who demonstrated that high carbohydrate diets contribute to greater central fat accumulation in a study of American adults. The reason is that carbohydrates and excess energy intake prefer to accumulate in the abdomen due to higher insulin levels stimulating fat storage in visceral fat cells, which are more metabolically active (McKeown et al., 2002; Després, 2012). These findings indicate that overconsumption of energy-dense foods, particularly those high in fat and carbohydrates, can disrupt the body's energy balance and promote the accumulation of body fat.

This study found an inverse relationship between anxiety levels and body composition. This finding is contrary to the work by Mather et al. (2009) and Luppino et al. (2010), who reported that higher levels of adiposity were linked to higher anxiety among adults in the United States and Brazil individually. The reason for the negative relationship perhaps is that higher adiposity can alter cortisol and serotonin levels, potentially reducing anxiety (Luppino et al., 2010). However, other psychological factors, such as disordered eating behaviors, may play an important role in shaping this relationship. For example, emotional eating and binge eating have been shown to influence body composition, and these behaviors are often associated with heightened anxiety levels (Luppino et al., 2010; Yu and Muehleman, 2023). Therefore, further research on the relationship between body composition and mental health is needed.

The study's findings showed a significant correlation between increased hunger before eating with higher body fat percentage and waist circumference. This aligns with research by Aloufi et al. (2022), which demonstrated that individuals with higher central fat experience greater hunger sensations before meals in a study involving Australian adults. Additionally, there was a significant positive association between BMI and waist circumference with hunger at 60 min after eating in this study. Rolls et al. (2004) found that individuals with a higher BMI are more likely to experience persistent hunger after meals in a study of American adults aged 25–55 years, which can lead to increased waist circumference. In individuals with increased central adiposity, elevated levels of ghrelin, known as the hunger hormone, stimulate appetite, while resistance to leptin reduces the brain's ability to signal fullness, leading to increased hunger sensations before meals (Morris and Rui, 2009; Briggs et al., 2014). Furthermore, higher BMI and central adiposity can disrupt the release of satiety hormones such as glucagon-like peptide-1 and cholecystokinin, resulting in prolonging hunger after meals (Vilsbøll et al., 2003; Strader and Woods, 2005). In addition, inflammation associated with elevated fat levels may further impair these signaling pathways, contributing to increased post-meal hunger (Thaler and Schwartz, 2010).

The findings indicate a significantly positive correlation between BMI with DTE before eating, at 30 min after eating, and at 60 min after eating among overweight and obese female college students. This is consistent with previous research. For instance, Burger et al. (2011) found that individuals with a higher BMI reported a greater desire to eat large portions of food before eating in 130 adults. Additionally, van Galen et al. (2023) demonstrated that a higher BMI exhibited a diminished brain response to nutrient intake, which could explain an increased DTE shortly after meals due to impaired nutrient sensing. The findings indicate that higher body fat percentages are associated with increased DTE both before eating and at 60 min after eating. This aligns with research by Blundell and Finlayson (2004), which found that higher body fat is linked to heightened appetite before eating and increased DTE shortly after meals. However, the correlation between lower DTE at 90 min and higher body fat percentage contrasts with a previous study by Wu et al. (2024), who found that a calorie-restricted diet combined with exercise significantly reduced body fat percentage in young women, along with a reduction in the DTE at later intervals post-meal. This study identifies a significantly positive correlation between waist circumference with DTE before eating and at 60 min after eating. A study by Carnell and Wardle (2008) shows a similar result, which is that waist circumference scores were associated with food cue responsiveness among children. Studies by Obradovic et al. (2021) have shown that higher levels of obesity are associated with elevated levels of leptin, which paradoxically might not effectively suppress appetite in obese individuals due to leptin resistance. However, there is an eventual leptin-mediated response despite initial resistance. This delayed response aligns with findings from Myers et al. (2010), who discussed how leptin signaling defects caused by fat can delay but not completely inhibit satiety signals, leading to a later disappearance of the feeling of DTE post-meal.

The result shows a positive correlation between the sensation of fullness immediately before eating and increased waist circumference. This finding contrasts with one previous study by Heymsfield et al. (2008), which found that a sensation of fullness before eating was negatively correlated with central adiposity. The sensation of fullness 30 min after eating is positively correlated with both body fat percentage and waist circumference, aligning with Liu et al. (2021), who observed prolonged satiety in individuals with higher adiposity due to slower gastric emptying in the postprandially early stage. Higher BMI, body fat percentage, and waist circumference are negatively correlated with fullness at 120 min after eating, which is consistent with findings by Holt et al. (2006) showing a prematurely reduced sense of satiety in obese individuals. Individuals with larger waist circumferences often experience insulin resistance, leading to higher circulating insulin levels, which can initially suppress appetite and create a sensation of fullness before eating (Deusdará et al., 2022). Additionally, individuals with higher central adiposity may have delayed gastric emptying. This delayed process increases postprandial fullness due to prolonged digestion times (Holt et al., 2006; Vijayvargiya et al., 2019). However, leptin and insulin resistance caused by obesity result in reduced efficacy of satiety hormones like peptide YY and cholecystokinin over time, ultimately leading to decreased fullness (Guyenet and Schwartz, 2012; Barakat et al., 2024).

The findings indicate that higher BMI, body fat percentage, and waist circumference are each individually associated with increased PFC before eating. This aligns with previous research by Fisher et al. (2003), which found that individuals with higher adiposity tend to anticipate higher food intake due to altered appetite regulation and energy needs. Additionally, higher BMI is associated with increased PFC at 30, 60, and 120 min after eating, consistent with the findings by Stunkard et al. (2004), who reported that individuals with higher BMI exhibit prolonged anticipatory eating behaviors post-meal. Like the previous reasons for the impact of obesity on hunger, leptin resistance and elevated ghrelin can also reduce the expected appetite before meals (Hernández Morante et al., 2020; Obradovic et al., 2021). Furthermore, higher BMI is associated with lower metabolic efficiency among overweight and obese individuals, which contributes to higher prospective food intake even after eating (Julibert et al., 2019; Johnson et al., 2021).

## 5 Limitation

This cross-sectional study aimed to establish associations, but integrating longitudinal and experimental studies could provide insights into causal relationships and long-term effects. For instance, weight management interventions influence appetite and dietary behaviors (Beaulieu et al., 2021), and longitudinal studies suggest bidirectional links between body weight and mental health (Zhang, 2021). Future research should investigate these causal pathways. Self-reported dietary assessments, commonly used in large cohort studies, are prone to bias but are cost-effective and applicable in large-scale research (Subar et al., 2015; Hébert, 2016). Future studies should employ more accurate techniques like weighed food records or doubly labeled water (Slimani et al., 2015; Ravelli and Schoeller, 2020). Given the impact of physical activity on body composition (Franklin, 2021; Whitfield et al., 2021), future studies should use objective tools like accelerometers to explore the activity-body composition relationship (Bartley et al., 2019). Lastly, as appetite regulation and mental health mechanisms vary with body composition across gender and age, future research should include diverse populations to improve generalizability (van Strien, 2018; Zhao et al., 2020; Diotaiuti et al., 2022; Nour et al., 2024).

## 6 Conclusion

The study found significant correlations between nutritional intake, appetite regulation, and mental health with body composition among overweight and obese female college students. Overall, body composition was positively correlated with energy, fat, and carbohydrate intake. In addition, body composition was positively correlated with hunger and PFC at different timepoints, as well as DTE before eating, at 30, and 60 min after eating, but negatively correlated with fullness at 120 min after eating, which is consistent with normal logic. Therefore, if overweight and obese female college students want to reduce their body composition, they need to care about the changes in energy intake, protein intake, fat intake, hunger, PFC, DTE before eating and during the early postprandial period, and fullness during the late postprandial period. However, the study found a negative correlation between body composition with anxiety and DTE at 90 min but a positive correlation with fullness at 0 and 30 min, challenging conventional wisdom. Future research should address these issues, along with limitations related to causal relationships, measurement accuracy, the relationship with physical activity, and population diversity.

## Data Availability

The raw data supporting the conclusions of this article will be made available by the authors, without undue reservation.
