# Supplementary material for: Relationships between nutritional intake, appetite regulation, and mental health with body composition among female college students with overweight and obesity
Source: Front Psychol. 2025 May 14;16:1465784. doi: 10.3389/fpsyg.2025.1465784 (PMC12120358; doi:10.3389/fpsyg.2025.1465784)
Supplement: Supplementary file 1 [file Supplementary_file_1.doc]

Table S1. Tests of normality

|  | Kolmogorov-Smirnova | | | Shapiro-Wilk | | |
| --- | --- | --- | --- | --- | --- | --- |
|  | Statistic | df | Sig. | Statistic | df | Sig. |
| BMI* | 0.206 | 72 | 0 | 0.813 | 72 | 0 |
| Body fat percentage | 0.071 | 72 | .200* | 0.964 | 72 | 0.04 |
| Waist circumference* | 0.13 | 72 | 0.004 | 0.927 | 72 | 0 |
| Energy intake | 0.059 | 72 | .200 | 0.971 | 72 | 0.1 |
| Protein intake* | 0.154 | 72 | 0 | 0.925 | 72 | 0 |
| Fat intake | 0.089 | 72 | .200 | 0.926 | 72 | 0 |
| Carbohydrate intake* | 0.149 | 72 | 0 | 0.894 | 72 | 0 |
| Depression* | 0.111 | 72 | 0.029 | 0.945 | 72 | 0.003 |
| Anxiety | 0.085 | 72 | .200 | 0.946 | 72 | 0.004 |
| Stress | 0.104 | 72 | 0.05 | 0.956 | 72 | 0.013 |
| Hunger 0mins | 0.101 | 72 | 0.067 | 0.934 | 72 | 0.001 |
| Hunger 30mins | 0.057 | 72 | .200 | 0.961 | 72 | 0.024 |
| Hunger 60mins* | 0.11 | 72 | 0.03 | 0.958 | 72 | 0.017 |
| Hunger 90mins | 0.088 | 72 | .200 | 0.949 | 72 | 0.005 |
| Hunger 120mins* | 0.15 | 72 | 0 | 0.932 | 72 | 0.001 |
| DTE 0mins* | 0.14 | 72 | 0.001 | 0.937 | 72 | 0.001 |
| DTE 30mins | 0.094 | 72 | 0.189 | 0.954 | 72 | 0.011 |
| DTE 60mins | 0.094 | 72 | 0.195 | 0.961 | 72 | 0.026 |
| DTE 90mins* | 0.232 | 72 | 0 | 0.853 | 72 | 0 |
| DTE 120mins* | 0.219 | 72 | 0 | 0.869 | 72 | 0 |
| Fullness_0mins* | 0.186 | 72 | 0 | 0.871 | 72 | 0 |
| Fullness_30mins* | 0.339 | 72 | 0 | 0.694 | 72 | 0 |
| Fullness_60mins | 0.147 | 72 | 0.071 | 0.898 | 72 | 0 |
| Fullness_90mins | 0.131 | 72 | 0.094 | 0.895 | 72 | 0 |
| Fullness_120mins* | 0.18 | 72 | 0 | 0.87 | 72 | 0 |
| PFC 0mins* | 0.154 | 72 | 0 | 0.922 | 72 | 0 |
| PFC 30mins* | 0.131 | 72 | 0.004 | 0.943 | 72 | 0.003 |
| PFC 60mins* | 0.112 | 72 | 0.026 | 0.963 | 72 | 0.032 |
| PFC 90mins* | 0.113 | 72 | 0.024 | 0.942 | 72 | 0.003 |
| PFC 120mins* | 0.186 | 72 | 0 | 0.916 | 72 | 0 |

**Both Kolmogorov-Smirnova and Shapiro-Wilk less than 0.05 means normal distribution*

Table S2. Descriptive statistics and Z-scores of variables

|  | Mean ± SD | Minimum | Maximum | Z-score |
| --- | --- | --- | --- | --- |
| BMI | 28.638 ± 3.233 | 25.02 | 33.66 | -1.119 to 1.553 |
| Body fat percentage | 27.855 ± 3.187 | 22.01 | 33.46 | -1.834 to 1.759 |
| Waist circumference | 92.836 ± 4.726 | 85.5 | 100.4 | -1.552 to 1.600 |
| Energy intake | 2155.635 ± 289.235 | 1655.31 | 2733.99 | -1.730 to 2.000 |
| Protein intake | 80.5 ± 21.216 | 44.27 | 116.89 | -1.708 to 1.715 |
| Fat intake | 70.123 ± 20.041 | 40.04 | 101.66 | -1.501 to 1.574 |
| Carbohydrate intake | 301.17 ± 61.392 | 206.46 | 393.79 | -1.543 to 1.509 |
| Depression | 7.68 ± 3.841 | 2 | 15 | -1.479 to 1.905 |
| Anxiety | 12.83 ± 5.679 | 4 | 23 | -1.555 to 1.790 |
| Stress | 16 ± 6.546 | 4 | 27 | -1.833 to 1.681 |
| Hunger 0mins | 42.385 ± 19.540 | 11.6 | 74.8 | -1.575 to 1.659 |
| Hunger 30mins | 14.839 ± 6.841 | 3.4 | 32.1 | -1.672 to 2.523 |
| Hunger 60mins | 27.506 ± 12.688 | 6.5 | 61.5 | -1.656 to 2.679 |
| Hunger 90mins | 31.668 ± 14.602 | 8.6 | 66.4 | -1.580 to 2.379 |
| Hunger 120mins | 34.942 ± 16.101 | 9.5 | 67.9 | -1.580 to 2.047 |
| DTE 0mins | 46.25 ± 16.622 | 13.9 | 82.6 | -1.946 to 2.187 |
| DTE 30mins | 21.351 ± 7.678 | 7.4 | 33.9 | -1.817 to 1.634 |
| DTE 60mins | 28.233 ± 10.142 | 9.2 | 50.7 | -1.877 to 2.215 |
| DTE 90mins | 36.275 ± 13.042 | 8.7 | 70.3 | -2.114 to 2.609 |
| DTE 120mins | 39.507 ± 14.205 | 11.3 | 76.8 | -1.986 to 2.625 |
| Fullness_0mins | 32.556± 16.628 | 13.3 | 65.2 | -1.158 to 1.963 |
| Fullness_30mins | 68.74 ± 35.122 | 5.1 | 98.9 | -1.812 to 0.859 |
| Fullness_60mins | 56.642 ± 28.938 | 15.1 | 98.7 | -1.436 to 1.453 |
| Fullness_90mins | 54.794 ± 28.005 | 18.1 | 98.7 | -1.310 to 1.568 |
| Fullness_120mins | 45.578 ± 23.290 | 13.4 | 78.2 | -1.382 to 1.401 |
| PFC 0mins | 52.578 ± 15.648 | 17.5 | 73.8 | -2.242 to 1.356 |
| PFC 30mins | 28.418 ± 8.459 | 16.3 | 47.7 | -1.433 to 2.279 |
| PFC 60mins | 34.049 ± 10.128 | 15.6 | 55.6 | -1.822 to 2.128 |
| PFC 90mins | 41.256 ± 12.282 | 20.3 | 61.6 | -1.706 to 1.656 |
| PFC 120mins | 46.678 ± 13.873 | 22.3 | 68.3 | -1.757 to 1.559 |


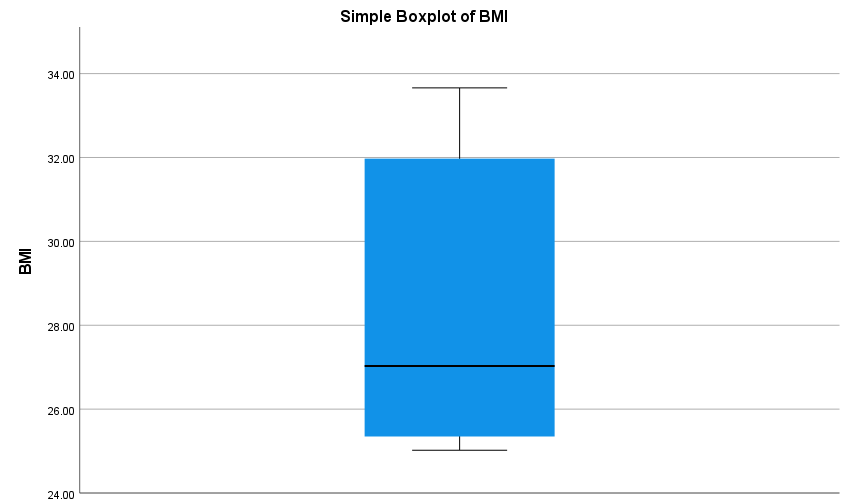


**Figure 1. Boxplot of BMI**


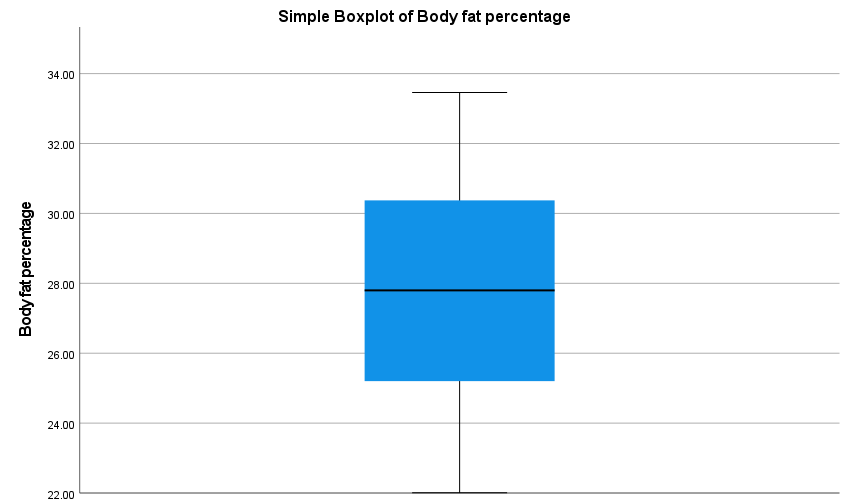


**Figure 2. Boxplot of body fat percentage**


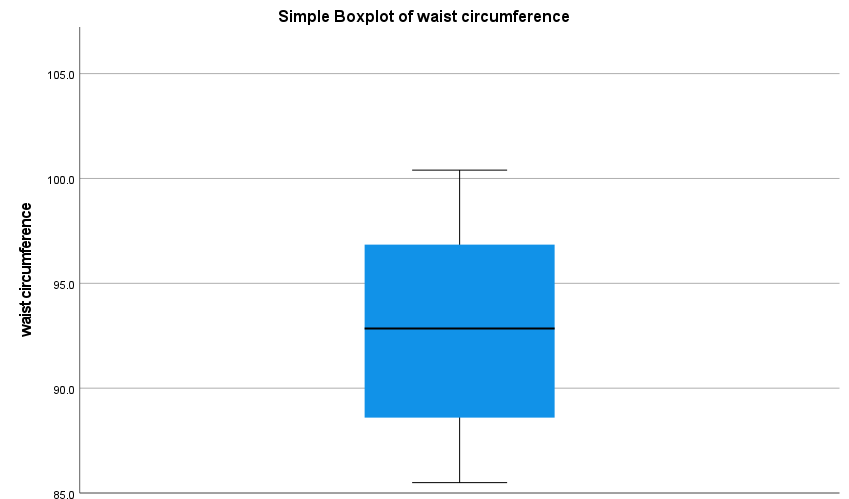


**Figure 3. Boxplot of waist circumference**

**
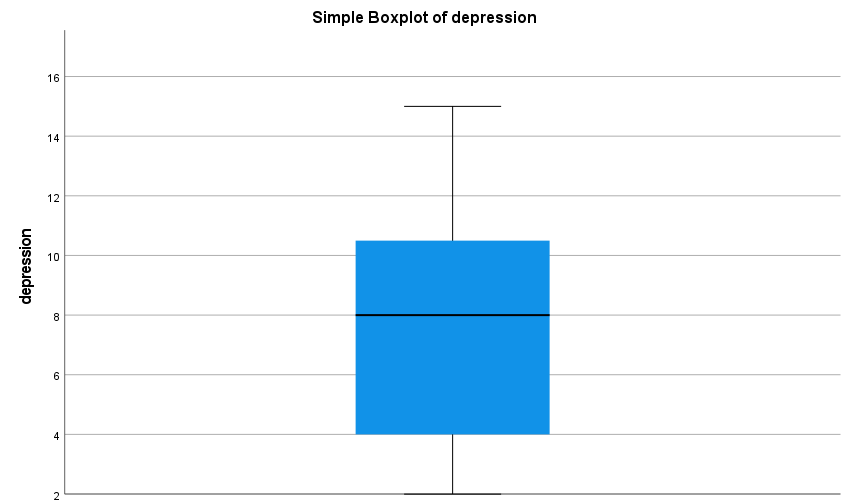
**

**Figure 4. Boxplot of depression**

**
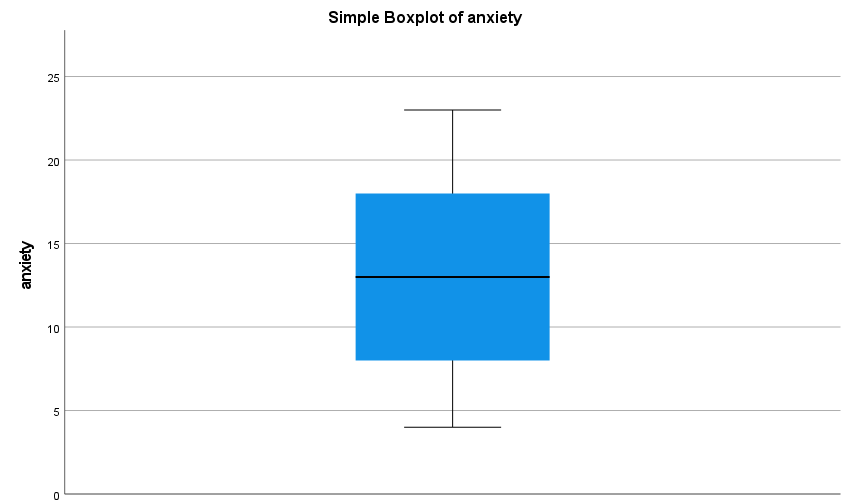
**

**Figure 5. Boxplot of anxiety**

**
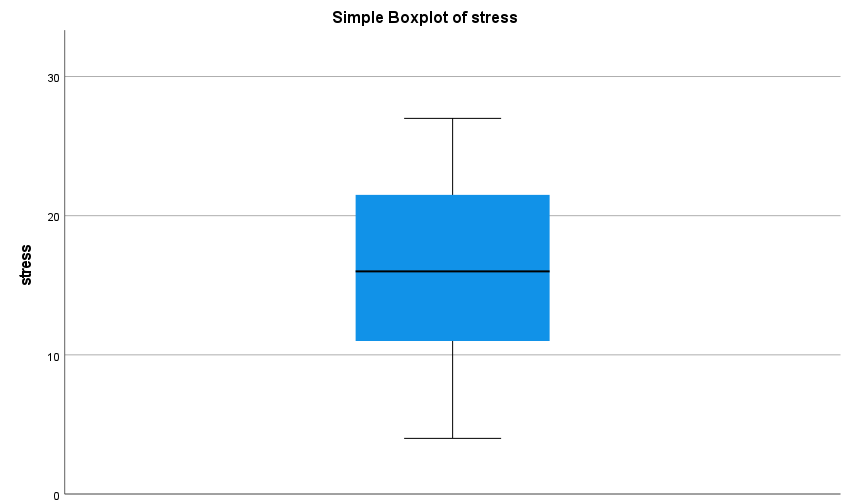
**

**Figure 6. Boxplot of stress**

**
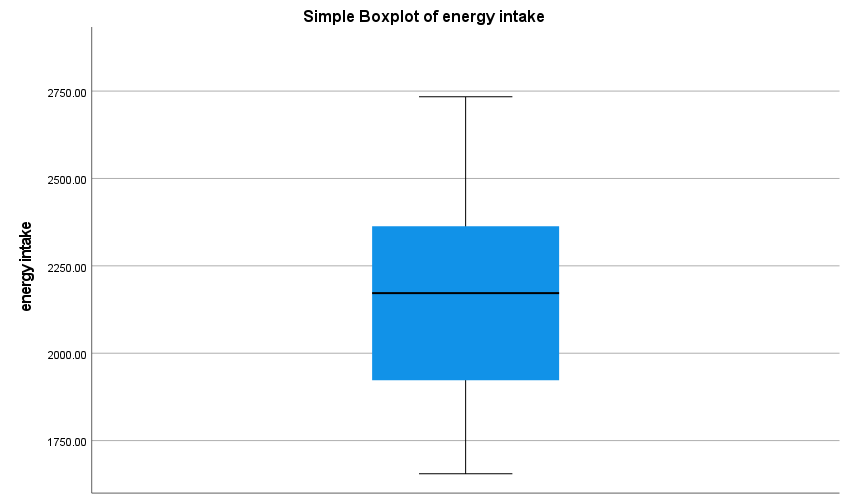
**

**Figure 7. Boxplot of energy intake**

**
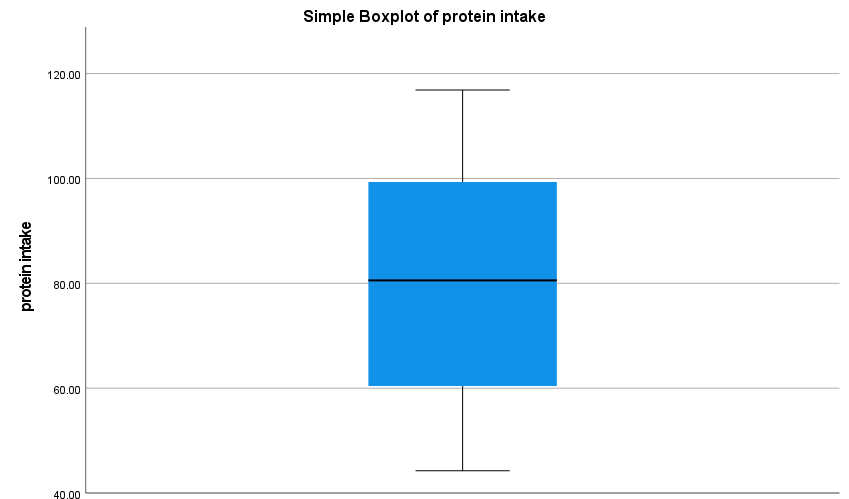
**

**Figure 8. Boxplot of protein intake**

**
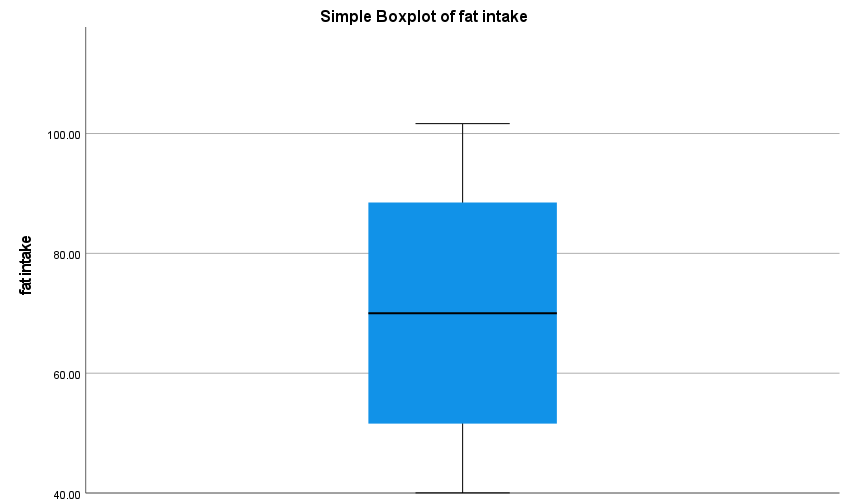
**

**Figure 9. Boxplot of fat intake**

**
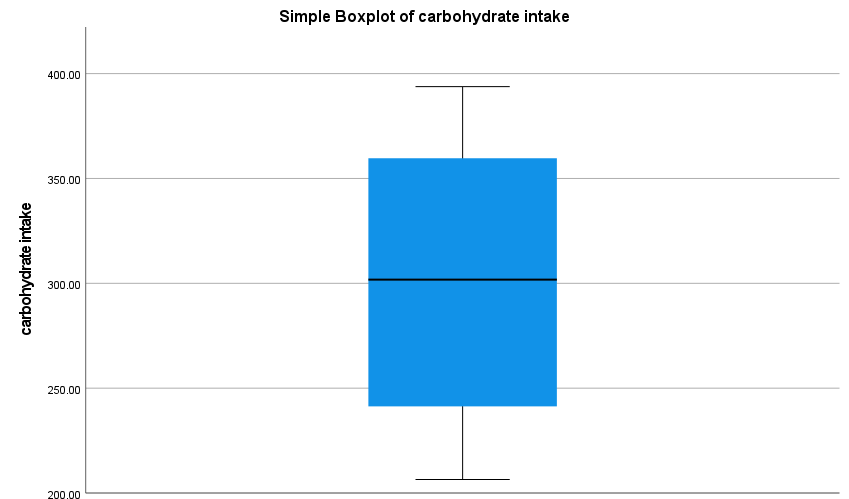
**

**Figure 10. Boxplot of carbohydrate intake**

**
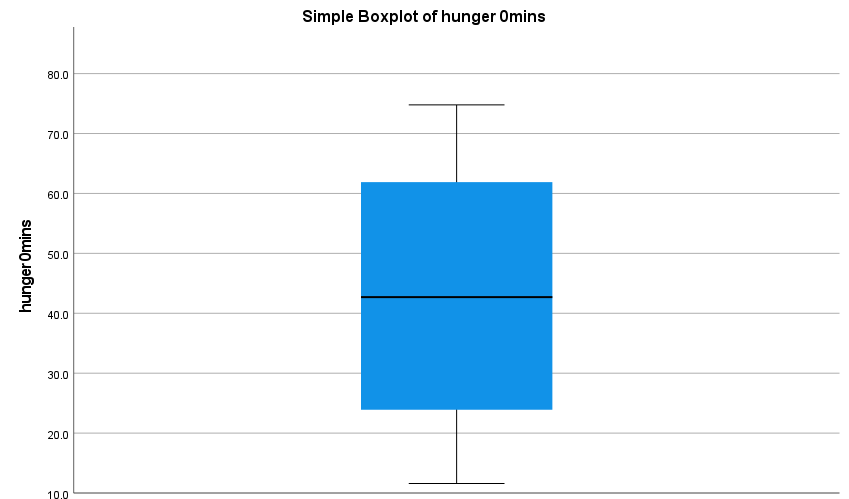
**

**Figure 11. Boxplot of hunger 0mins**

**
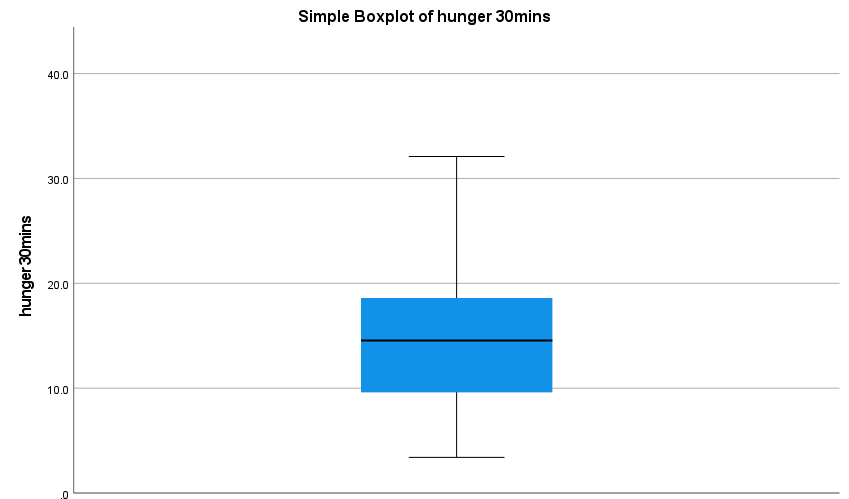
**

**Figure 12. Boxplot of hunger 30mins**

**
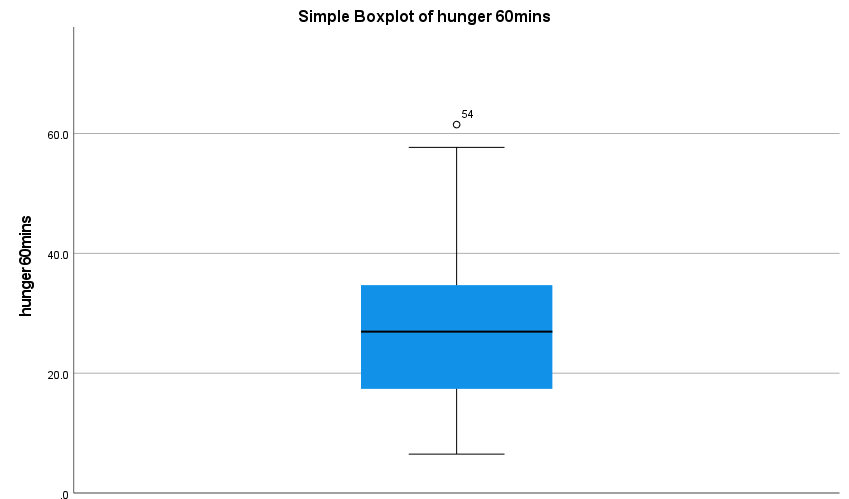
**

**Figure 13. Boxplot of hunger 60mins**

**
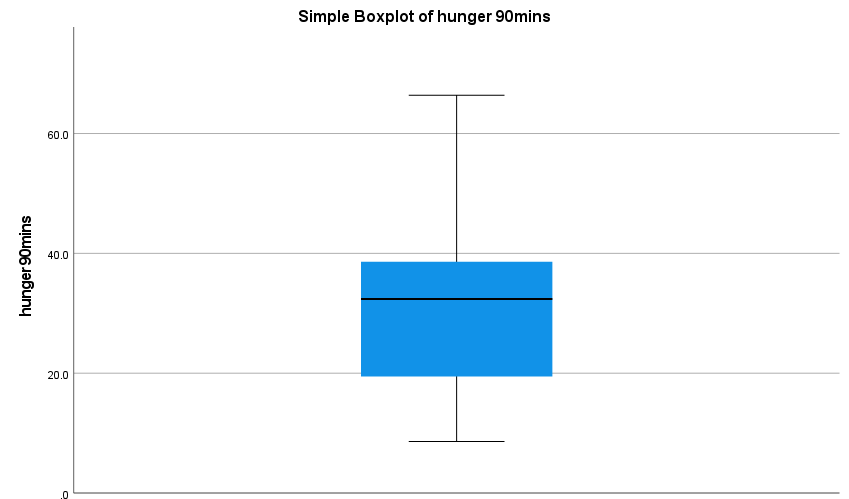
**

**Figure 14. Boxplot of hunger 90mins**

**
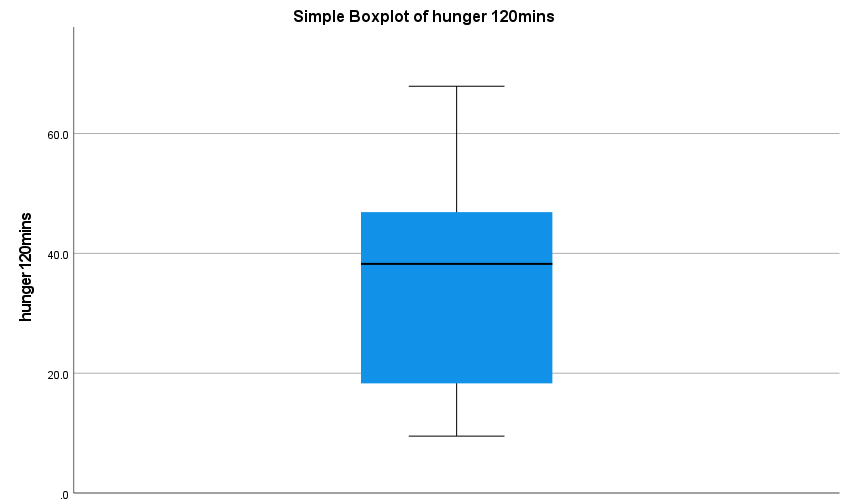
**

**Figure 15. Boxplot of hunger 120mins**

**
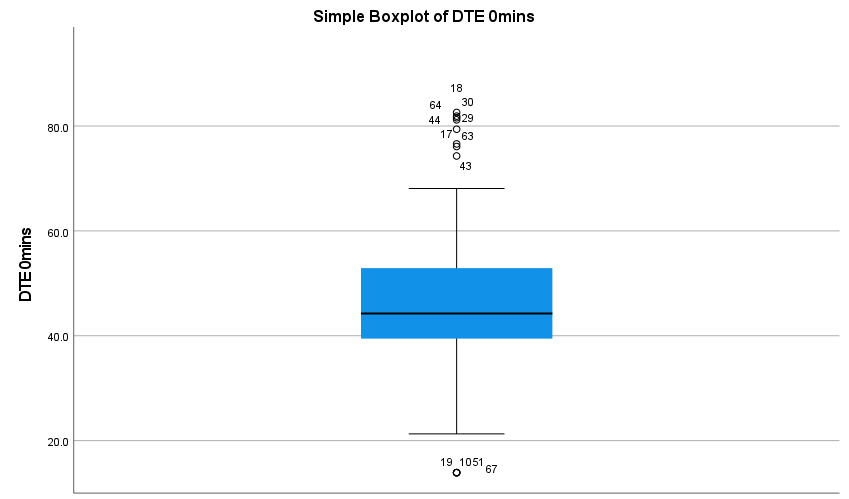
**

**Figure 16. Boxplot of DTE 0mins**

**
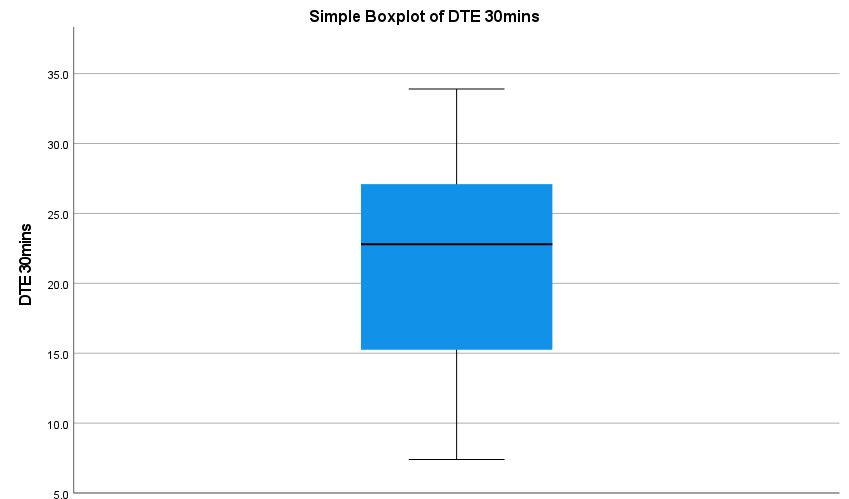
**

**Figure 17. Boxplot of DTE 30mins**

**
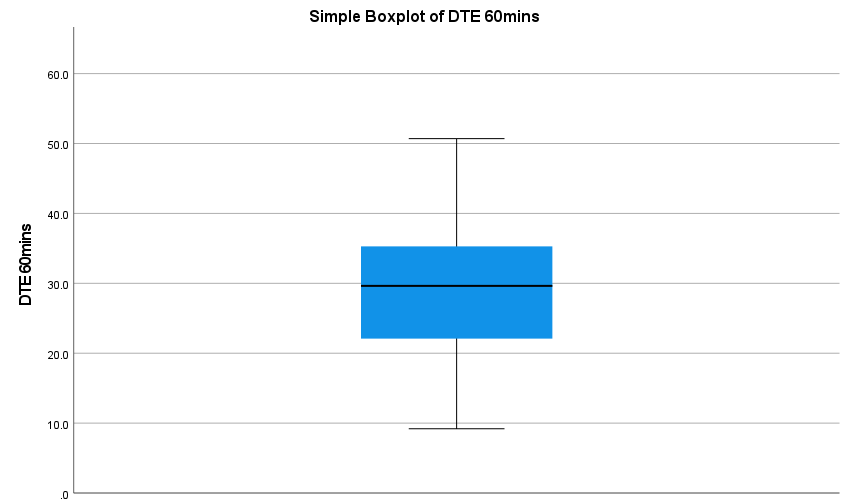
**

**Figure 18. Boxplot of DTE 60mins**

**
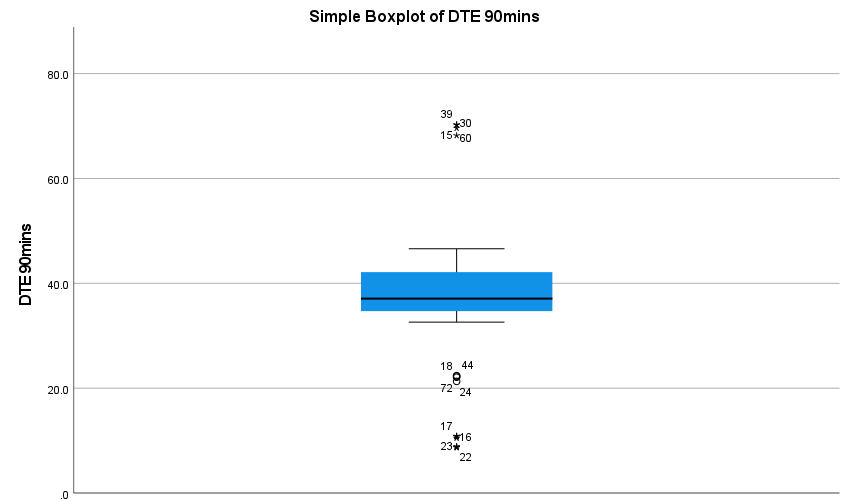
**

**Figure 19. Boxplot of DTE 90mins**

**
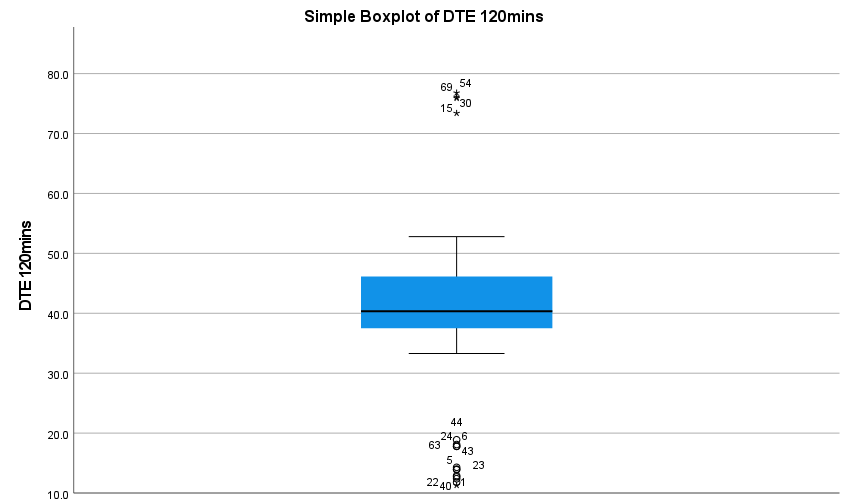
**

**Figure 20. Boxplot of DTE 120mins**

**
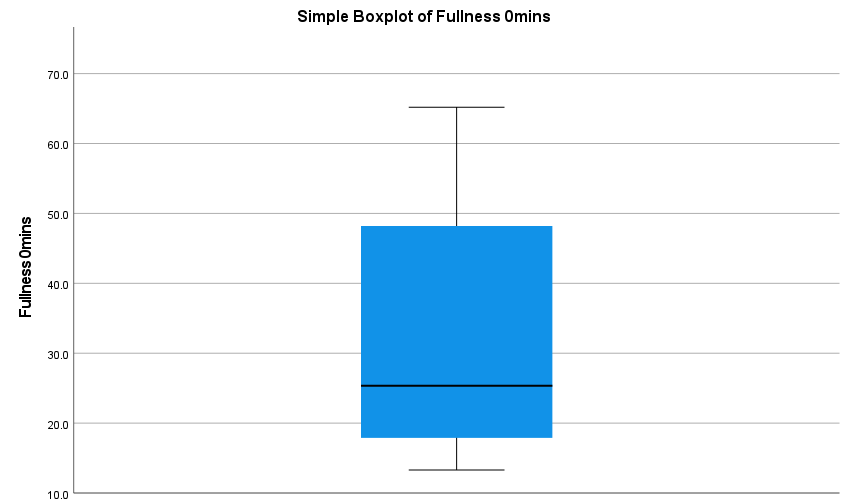
**

**Figure 21. Boxplot of fullness 0mins**

**
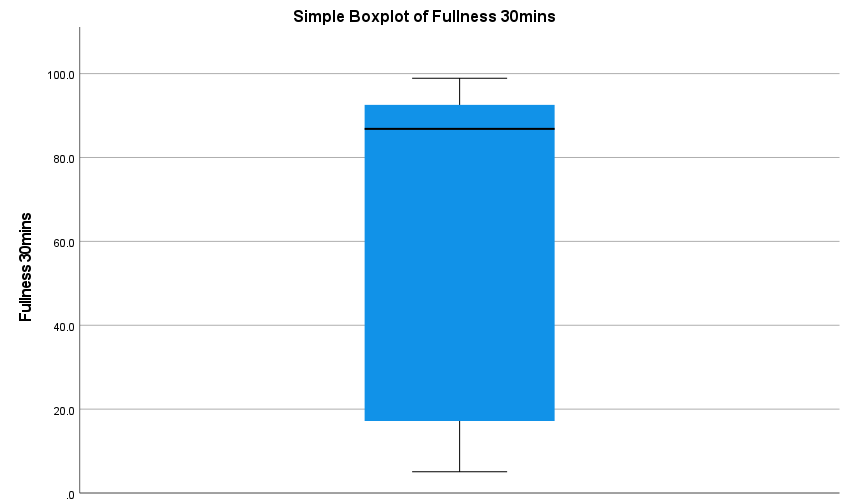
**

**Figure 22. Boxplot of fullness 30mins**

**
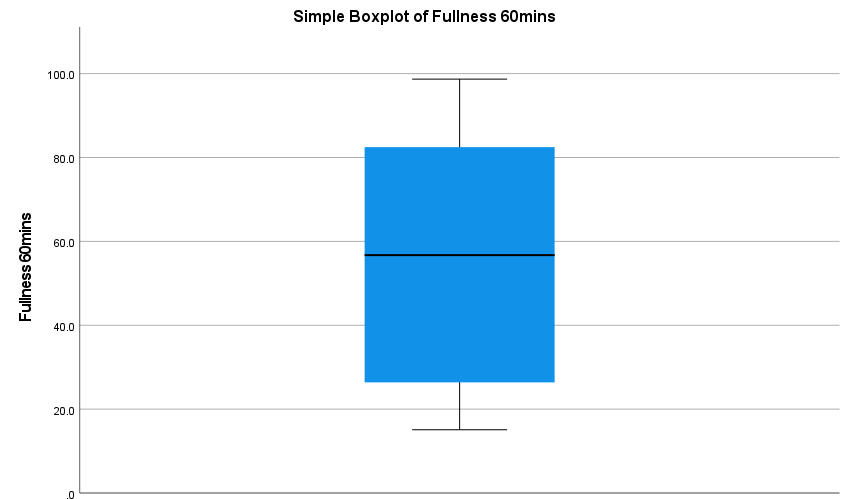
**

**Figure 23. Boxplot of fullness 60mins**

**
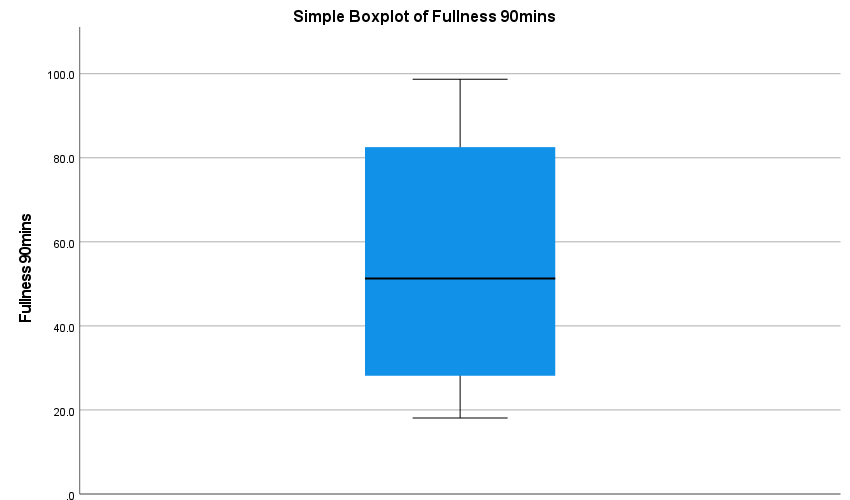
**

**Figure 24. Boxplot of fullness 90mins**

**
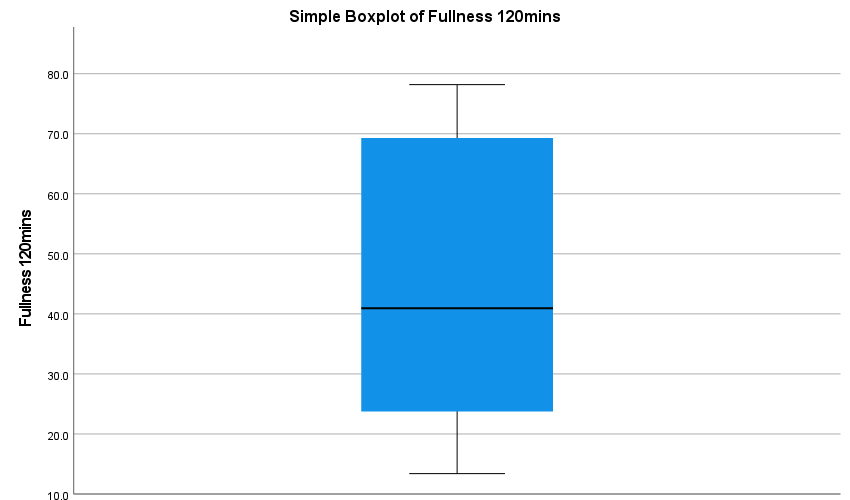
**

**Figure 25. Boxplot of fullness 120mins**

**
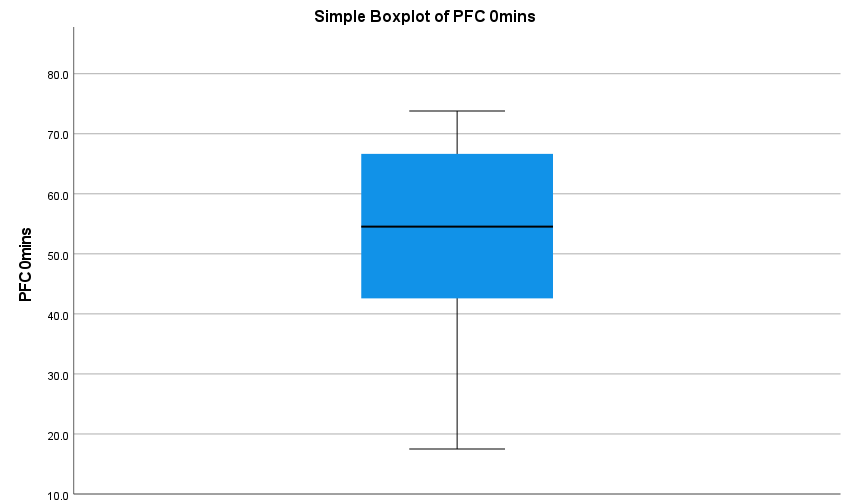
**

**Figure 26. Boxplot of PFC 0mins**

**
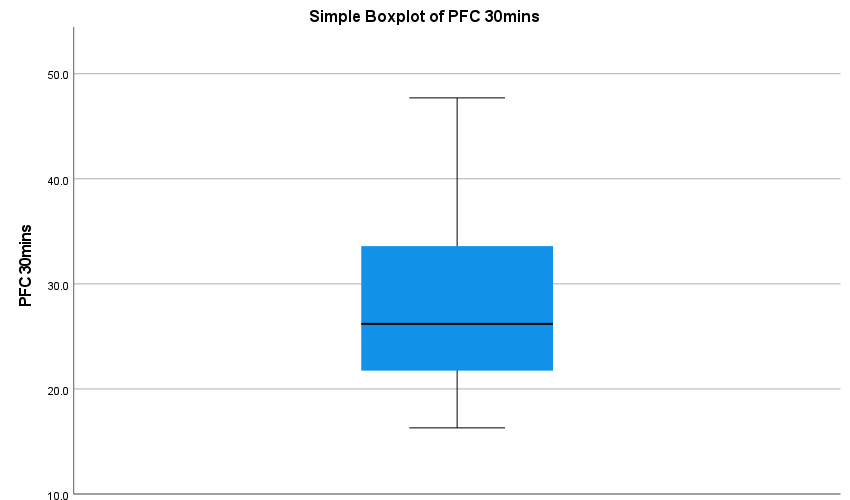
**

**Figure 27. Boxplot of PFC 30mins**

**
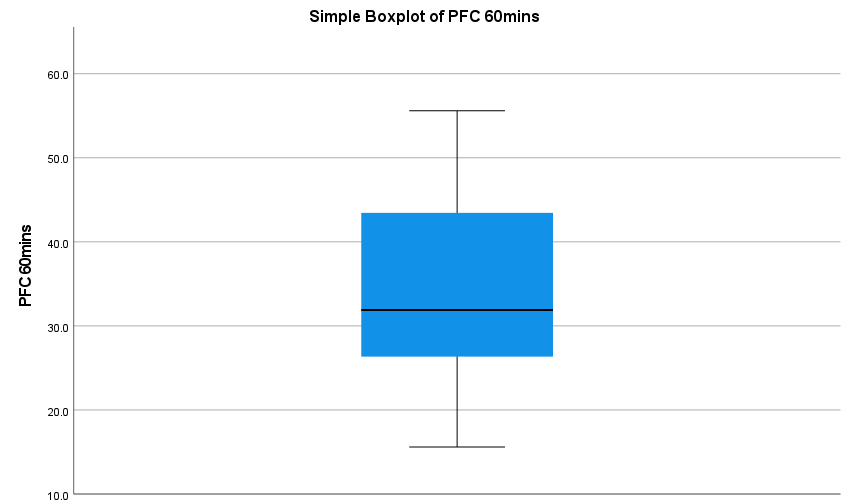
**

**Figure 28. Boxplot of PFC 60mins**

**
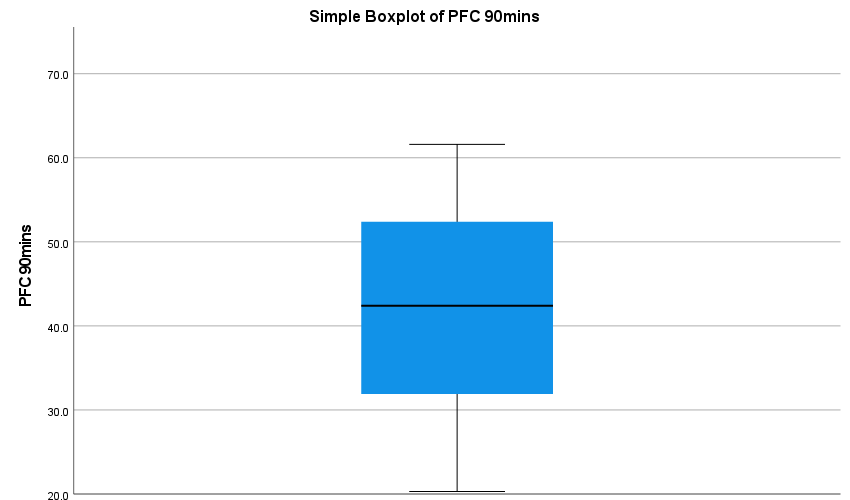
**

**Figure 29. Boxplot of PFC 90mins**

**
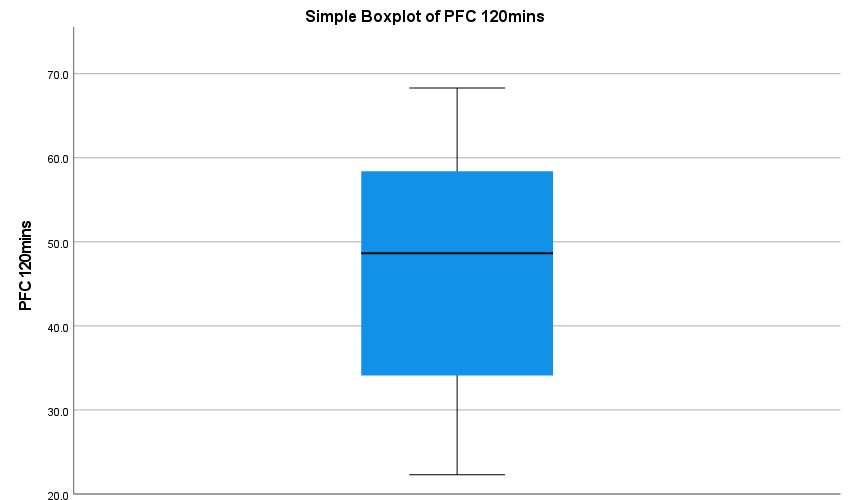
**

**Figure 30. Boxplot of PFC 120mins**
